# Supplementary figures and images for: Efficient wastewater sample filtration improves the detection of SARS-CoV-2 variants: An extensive analysis based on sequencing parameters
Source: PLoS One. 2024 May 24;19(5):e0304158. doi: 10.1371/journal.pone.0304158 (PMC11125551; doi:10.1371/journal.pone.0304158)

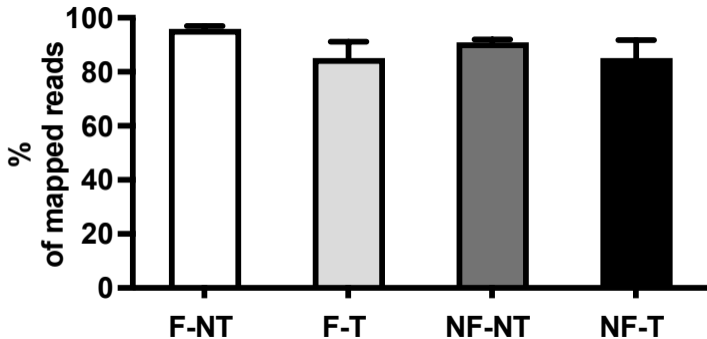

Supplement: S2 Fig — Mapped reads refer to those reads that align directly to regions on the SARS-CoV-2 reference genome. Bar plots represent the mean and standard deviation. (PDF) [file pone.0304158.s002.pdf]
