# Supplementary material for: Efficient wastewater sample filtration improves the detection of SARS-CoV-2 variants: An extensive analysis based on sequencing parameters
Source: PLoS One. 2024 May 24;19(5):e0304158. doi: 10.1371/journal.pone.0304158 (PMC11125551; doi:10.1371/journal.pone.0304158)
Supplement: S1 Table — (PDF) [file pone.0304158.s003.pdf]

| | | **RNA Treatment: DNase I / RNA-Clean-up KIT** | |
|---|---|---|---|
| | | YES | NO |
| **WW Filtrations: 0.45 µm + 0.2 µm subsequently** | YES | 3 samples (F-T) | 3 samples (F-NT) |
| | NO | 3 samples (NF-T) | 3 samples (NF-NT) |
