# Supplementary material for: Efficient wastewater sample filtration improves the detection of SARS-CoV-2 variants: An extensive analysis based on sequencing parameters
Source: PLoS One. 2024 May 24;19(5):e0304158. doi: 10.1371/journal.pone.0304158 (PMC11125551; doi:10.1371/journal.pone.0304158)
Supplement: S2 Table — (PDF) [file pone.0304158.s004.pdf]

		Treatment:<br>DNase / RNA-Clean-up			
		YES		NO	
**Filtrations: (0.45µm+0.2 µm subsequently)**	YES	WWTP1_FT<br>(Ct: 30.91; Cp/µl:75.57)	Average Cp/µl:<br>56.91±16.79	WWTP1_F<br>(Ct:30.98; Cp/µl: 71.71)	Average Cp/µl:<br>46.38±19.28
		WWTP2_FT<br>(Ct:31.23; Cp/µl: 60.31)		WWTP2_F<br>(Ct:31.71; Cp/µl: 42.46)	
		WWTP3_FT<br>(Ct:31.98, Cp/µl: 34.86)		WWTP3_F<br>(Ct:32.44; Cp/µl: 24.98)	
	NO	WWTP1_NFT<br>(Ct:30.52; Cp/µl: 100.38)	Average Cp/µl:<br>71.27±20.75	WWTP1_NF<br>(Ct:30.81; Cp/µl:83.87)	Average Cp/µl:<br>56.04±26.63
		WWTP2_NFT<br>(Ct:31.23; Cp/µl: 59.92)		WWTP2_NF<br>(Ct:31.38; Cp/µl:53.45)	
		WWTP3_NFT<br>(Ct:31.39; Cp/µl: 53.52)		WWTP3_NF<br>(Ct:32.16; Cp/µl:30.79)	
