# Supplementary material for: Efficient wastewater sample filtration improves the detection of SARS-CoV-2 variants: An extensive analysis based on sequencing parameters
Source: PLoS One. 2024 May 24;19(5):e0304158. doi: 10.1371/journal.pone.0304158 (PMC11125551; doi:10.1371/journal.pone.0304158)
Supplement: S3 Table — (PDF) [file pone.0304158.s005.pdf]

	WWTP-1 (bp)	WWTP-2 (bp)	WWTP-3 (bp)	Average (bp)
**F-NT**	386	380	378	381.3
**F-T**	381	377	353	370.3
**NF-NT**	374	372	377	374.3
**NF-T**	375	381	354	370
