# Supplementary material for: Efficient wastewater sample filtration improves the detection of SARS-CoV-2 variants: An extensive analysis based on sequencing parameters
Source: PLoS One. 2024 May 24;19(5):e0304158. doi: 10.1371/journal.pone.0304158 (PMC11125551; doi:10.1371/journal.pone.0304158)
Supplement: S4 Table — A Warning is issued if the lower quartile for any base is less than 10, or if the median for any base is less than 25. A Failure is reported if the lower quartile for any base is less than 5 or if the median for any base is less than 20. (PDF) [file pone.0304158.s006.pdf]

	Passed	Warning	Failure
WWTP1_F-NT_R1	X		
WWTP1_F-NT_R2	X		
WWTP2_F-NT_R1	X		
WWTP2_F-NT_R2	X		
WWTP3_F-NT_R1	X		
WWTP3_F-NT_R2	X		
WWTP1_F-T_R1	X		
WWTP1_F-T_R2	X		
WWTP2_F-T_R1	X		
WWTP2_F-T_R2	X		
WWTP3_F-T_R1		X	
WWTP3_F-T_R2			X
WWTP1_NF-NT_R1	X		
WWTP1_NF-NT_R2		X	
WWTP2_NF-NT_R1		X	
WWTP2_NF-NT_R2			X
WWTP3_NF-NT_R1	X		
WWTP3_NF-NT_R2	X		
WWTP1_NF-T R1	X		
WWTP1_NF-T R2			X
WWTP2_NF-T R1	X		
WWTP2_NF-T R2			X
WWTP3_NF-T_R1			X
WWTP3_NF-T_R2			X
