# Supplementary material for: Efficient wastewater sample filtration improves the detection of SARS-CoV-2 variants: An extensive analysis based on sequencing parameters
Source: PLoS One. 2024 May 24;19(5):e0304158. doi: 10.1371/journal.pone.0304158 (PMC11125551; doi:10.1371/journal.pone.0304158)
Supplement: S1 Fig — Warning: a warning is issued if the lower quartile for any base is less than 10, or if the median for any base is less than 25. Failure: This module raises a failure if the lower quartile for any base is less than 5 or if the median for any base is less than 20. (PDF) [file pone.0304158.s001.pdf]

		WWTP1	WWTP2	WWTP3
F	R1	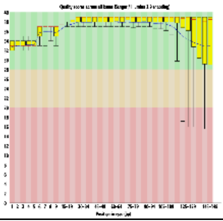	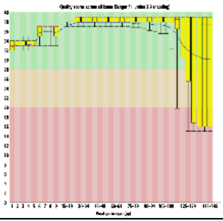	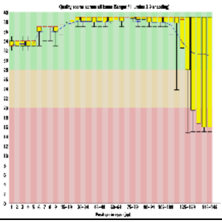
	R2	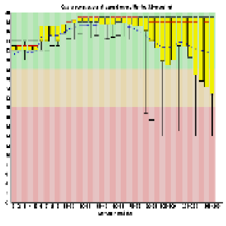	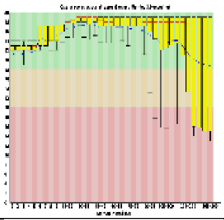	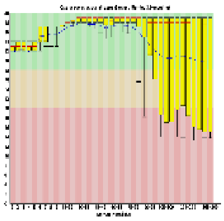
F - T	R1	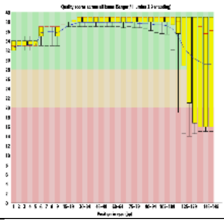	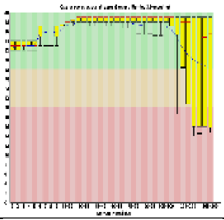	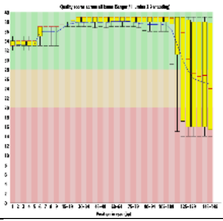
	R2	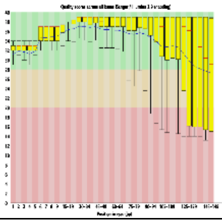	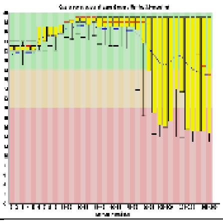	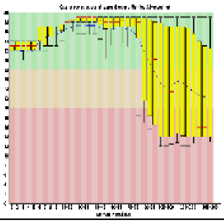
NF - NT	R1	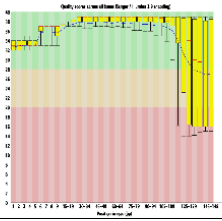	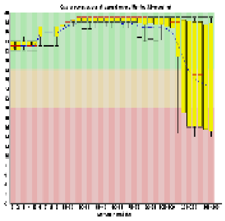	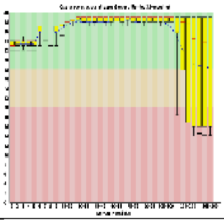
	R2	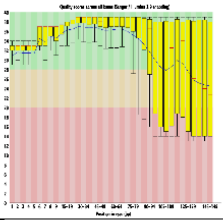	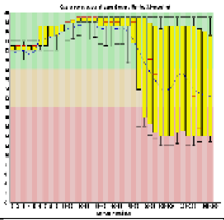	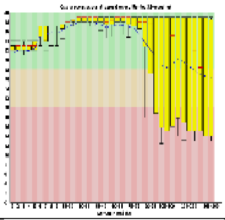
NF - T	R1	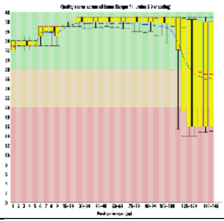	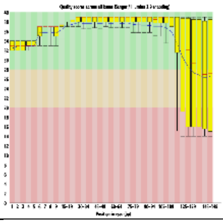	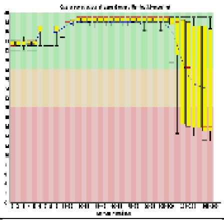
	R2	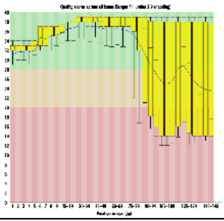	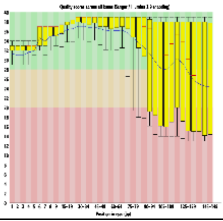	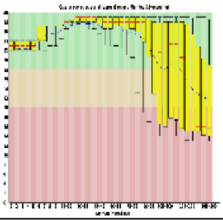
