# Supplementary material for: Efficient wastewater sample filtration improves the detection of SARS-CoV-2 variants: An extensive analysis based on sequencing parameters
Source: PLoS One. 2024 May 24;19(5):e0304158. doi: 10.1371/journal.pone.0304158 (PMC11125551; doi:10.1371/journal.pone.0304158)
Supplement: Attachment — Submitted filename: Response to Reviewers.docx [file pone.0304158.s007.docx]

**Dear Dr. Giandhari,**

**Thank you for your letter and the opportunity to submit the revised manuscript. We have addressed all the points raised in the editor’s and reviewers’ comments (bold sentences). Each point has been addressed, with changes tracked in the accompanying manuscript. We appreciate the chance to revise and submit our manuscript.**

**1**. Please ensure that your manuscript meets PLOS ONE's style requirements, including those for file naming. The PLOS ONE style templates can be found at <https://journals.plos.org/plosone/s/file?id=wjVg/PLOSOne_formatting_sample_main_body.pdf> and <https://journals.plos.org/plosone/s/file?id=ba62/PLOSOne_formatting_sample_title_authors_affiliations.pdf>

**We revised the style requirements.**

**2**. Thank you for stating the following financial disclosure:

“Arpa Piemonte received no specific funding for this work.”

At this time, please address the following queries:

**a)** Please clarify the sources of funding (financial or material support) for your study. List the grants or organizations that supported your study, including funding received from your institution.

**b)** State what role the funders took in the study. If the funders had no role in your study, please state: “The funders had no role in study design, data collection and analysis, decision to publish, or preparation of the manuscript.”

**c)** If any authors received a salary from any of your funders, please state which authors and which funders.

**d)** If you did not receive any funding for this study, please state: “The authors received no specific funding for this work.”

Please include your amended statements within your cover letter; we will change the online submission form on your behalf.

**We thank the editor for these comments. We have amended and included the Financial Disclosure with these sentences: *“Arpa Piemonte received no specific funding for this work. Professor DL was supported by EU funding within the MUR PNRR Extended Partnership initiative on Emerging Infectious Diseases (Project no. PE00000007, INF-ACT).*** ***The funders had no role in study design, data collection and analysis, decision to publish, or preparation of the manuscript.”.***

**3**. Thank you for stating the following in the Acknowledgments Section of your manuscript:

“The authors acknowledge the financial support received from Assicurazioni Generali and Intesa San Paolo to realize the Regional Centre of Molecular Biology run by Arpa Piemonte (Environmental Protection Agency of Piedmont) in La Loggia, Torino, Italy. This research was supported by EU funding within the MUR PNRR Extended Partnership initiative on Emerging Infectious Diseases (Project no. PE00000007, INF-ACT) to DL.”

We note that you have provided funding information that is not currently declared in your Funding Statement. However, funding information should not appear in the Acknowledgments section or other areas of your manuscript. We will only publish funding information present in the Funding Statement section of the online submission form.

Please remove any funding-related text from the manuscript and let us know how you would like to update your Funding Statement. Currently, your Funding Statement reads as follows:

“Arpa Piemonte received no specific funding for this work.”

Please include your amended statements within your cover letter; we will change the online submission form on your behalf.

**We have amended the Acknowledgement section: “*Arpa Piemonte acknowledges the financial support received from Assicurazioni Generali and Intesa San Paolo to realize the Regional Centre of Molecular Biology in La Loggia, Torino, Italy.”***

**4.** We note that you have stated that you will provide repository information for your data at acceptance. Should your manuscript be accepted for publication, we will hold it until you provide the relevant accession numbers or DOIs necessary to access your data. If you wish to make changes to your Data Availability statement, please describe these changes in your cover letter and we will update your Data Availability statement to reflect the information you provide.

**We commit to providing the accession numbers upon acceptance of the manuscript for publication.**

**5.** We notice that your S2Fig is uploaded with the file type 'Figure'. Please amend the file type to 'Supporting Information'. Please ensure that each Supporting Information file has a legend listed in the manuscript after the references list.

**We have removed S2 Fig as suggested by reviewer #2**

**Reviewer #1**: The authors focused on the use of wastewater surveillance to not only monitor the level of infection within a population but also to use for the early detection of variants. This paper is a response to recommendations from the European Union Commission to establish an approach to establish WW surveillance of SARS-CoV-2 and its variants. The authors aimed to obtain high-quality sequencing data to allow variants detection and compared for sample conditions and evaluated the best condition by assessing several sequencing parameters. The authors provided an overview of sequencing parameters to consider when the final goal is to detect variants.

It is important to use WW to monitor the level of infection within a population, but WW is also needed for the early detection of variants as the clinical sequencing data will continue to focus on symptomatic patients. Surveillance, quantification, sequencing, and comparison of results is intricate as wastewater is a complex matrix. Therefore, this study is valuable and important. However, the statistical analysis of the results needs to be revised and explained further before the proposed conclusions can be validated. Additionally, the high viral load found in the samples studied make it difficult to extrapolate the proposed results to a more “normal” scenario. The following issues need to be addressed before publication:

Issues


1- Lines 240-245. To compare the read frequencies differences between the four experimental conditions for three biological samples, the authors use the student’s t-test. There are requirements for applying the t-test: 1- normal distribution of the data obtained however, the sample number is too low to predict normal distribution.2- is used for pairwise comparisons, however four conditions are being compared. ANOVA for multiple comparisons is more suitable. After using a proper statistic test the significance of the differences detected should be reassessed.

**We thank the reviewer for these appropriate comments. We performed two-way ANOVA statistical tests for all the experiments but omitted to include the results in the manuscript. We have now stated ANOVA results for every figure of the manuscript. We revised statistical analysis throughout the manuscript. To assess the assumption of normal distribution of the data, we perform visual inspection analyses (histogram and Quantile-Quantile (QQ) plot) and statistical analysis (Shapiro-Wilk test) for each experiment.**

**The assumption of normal distribution was statistically significant for each figure in the manuscript, so two-way ANOVA and t-test were applied.**

**For lines 240-245 (now 253-259) we compared the copies input number for the different conditions. ANOVA and t-test results have been included in the manuscript as follows:**

**- line 257: Two-way ANOVA (* = p ≤ 0.05), has been added.**

**- in line 172 paragraph 2.7 “statistical analysis” has been edited: “The normal distribution of the data was assessed by visual inspection analyses (histogram and Quantile-Quantile (QQ) plot) and statistical analysis (Shapiro-Wilk test).** **The significance of the data was calculated using two-way ANOVA (Analysis of variance) and paired Student's t-test.”**

2- The authors should follow the MIQE guidelines for reporting their qPCR results. The standard curve, ranging from 102 copies (cp)/μL to 0.5 cp/μL that is generated by serial 10-fold dilution does not provide enough points.

**We made a mistake in reporting the data relative to the standard curve. The highest point of the standard curve is 10^3^. We corrected the value in line 134**

3- The authors do not provide an explanation of why the DNAse treatment and concentration of RNA in filtered samples increases the % of reads corresponding to Bacteria.
**We thank the reviewer for making this point.**

**After reviewing the statistics, we have removed the sentence about the concentration step being the reason for the increase of bacteria reads %.**

Minor issues
1- Line 129 describes 2 μL of TNA or purified RNA from each extracted sample was used for the one-step RT-qPCR. However, line 136 mentions 5μL of TNA/RNA for each WW sample and standard point.

**We have made a mistake. The correct volume is 5 μL (line 138).**

2- Line 170 mentions ANOVA Analysis of variance but the results mention t-test significance.

**We have stated the ANOVA results in the caption of each figure in the manuscript.**

**Reviewer #2**: The authors have conducted a study comparing methods for preparation of wastewater samples for amplicon sequencing of SARS-CoV-2, in particular assessing how the quality of sequencing data is impacted by prefiltering wastewater and implementing a step for purifying and concentrating extracted RNA. The authors conclude that filtration of wastewater samples leads to improved specificity and coverage of SARS-CoV-2 sequencing data, while treatment of RNA samples reduced the percentage of sequencing reads aligned to SARS-CoV-2 but had little impact on other sequencing metrics.
Although the experimental design and metrics used to assess SARS-CoV-2 sequencing quality were valid, the statistical methods used in analysis require improvement and clarification to draw valid conclusions. In particular, the statistical tests need to take into account the fact that samples exposed to the different treatments were derived from the same three wastewater samples and should therefore be treated as matched samples. Authors should also carry out comprehensive statistical tests (e.g. two-way ANOVA) to compare all the treatments together before implementing pairwise comparisons as they have currently done. Furthermore, the manuscript contains multiple linguistics errors and typos which need addressing, possibly through the use of a copyeditor.
Considering this, I recommend that this manuscript is publishable with major revisions.

Major issues
- The statistical methods used in data analysis require revision and clarification. Currently, the authors have stated in the methods (line 169-170) that they used “Student's t-test and ANOVA Analysis of variance” for statistical analysis. However, there is no mention of the results of ANOVA in the manuscript associated with any of the figures, making it unclear whether these tests were carried out. Furthermore, as mentioned above, the replicates within each treatment were derived from the same three wastewater samples, meaning statistical tests should consider that these replicates are matched between treatments. Finally, the two treatment variables that the authors are trying to determine the effects of (filtration and RNA treatment) are applied consecutively to these samples and are therefore nested, meaning these shouldn’t be considered as four separate treatments of one independent variable. The impact of these variables should be assessed by two-way ANOVA (or non-parametric equivalent, depending if parametric assumptions are met) to determine the overall effect of the two variables and any interaction between them.

**We thank the reviewer for these appropriate comments. We have revised the statistical analysis throughout the manuscript. We have considered the samples as matched, as the same sample is exposed to the different treatments, as the reviewer points out. We performed two-way ANOVA statistical tests for all the experiments but omitted to include the results in the manuscript. We have now reported the ANOVA results in every caption of the manuscript.**

**- in lines 172-175: Paragraph 2.7 “statistical analysis” was amended to read: “The normal distribution of the data was assessed by visual inspection analyses (histogram and Quantile-Quantile (QQ) plot) and statistical analysis (Shapiro-Wilk test).** **The significance of the data was calculated using two-way ANOVA (Analysis of variance) and paired Student's t-test.”**

- The manuscript contains grammatical, linguistic and typographical errors, which require revision. I have detailed a few of these in the minor issues. I recommend that the authors could consult a copyeditor to resolve this.

**We thank the reviewer for pointing out the typographical errors. The manuscript has been grammatically revised.**

Minor Issues
- Line 33 – it is stated that previous studies are “ignoring” wastewater filtering and RNA purification, despite these factors being addressed in multiple studies (as referenced). Consider revising this statement.

**We agree with the reviewer’s comment. We amend line 33. The sentence reads as follows: “Therefore, different groups have approached the issue with different strategies, mainly focusing on WW concentration methods, but only a few groups highlight the importance of prefiltering WW samples and/or purification of RNA samples.”**

- Line 35 – more information should be given on the treatments i.e i) filtering wastewater and i) DNAse treatment, purification and concentration of RNA.

**We thank the reviewer for asking us to clarify this sentence. We amended the sentence from line 34 to read: “Aiming to obtain high-quality sequencing data** **allowing variants detection, we compared four sample conditions generated from the treatment of: i) WW samples by WW filtration and ii) the extracted RNA by DNAse treatment, purification and concentration of the extracted RNA.”**

- Line 54 – please define “WW” again in the main text.

**We have defined WW abbreviation in line 55.**

- Line 54 – revise grammar in “was proposed as an epidemiologic tool approach…”

**In lines 53-55 the sentence has been revised with: “In the midst of the pandemic caused by Severe Acute Respiratory Syndrome Coronavirus - 2 (SARS-CoV-2), Wastewater Based Epidemiology (WBE) was proposed as an epidemiological tool approach for wastewater (WW) surveillance.**

- Line 59 – “transmission among asymptomatic has also” needs amending e.g. asymptomatic hosts.

**We added the word “hosts” in line 60.**

- Line 78-79 – The statement “amplicon sequencing for viral WGS is a preferable approach to library preparation” requires revision, as amplicon sequencing involves library preparation. Please revise this to state which method of library preparation you are comparing amplicon sequencing to.

**We revised the sentence: “Whole-genome sequencing (WGS) of viruses has become a powerful tool for studying emerging infectious diseases while monitoring public health and amplicon sequencing for viral WGS is the preferable approach for library preparation since it is simple, cost-effective and sensitive for detecting genomes from samples with low viral loads.” in lines 78-81.**

- Line 82-83 – Please revise the grammar in “but not many monitoring have been”

**We revised the grammar in lines 84.**

- Authors names need to be added where references are used as part of sentences in the text, or sentence structure amended accordingly. This occurs in multiple locations in the manuscript e.g. Line 88 “…studies conducted by 21-22 showed…”.

**We amended the sentences according to this comment.**

- Line 97 – amend typo “ae”.

**We amended the typo.**

- Line 116 – Please clarify whether the centrifugation step mentioned in this line is an additional step to the centrifugation described in line 107, or a repeated description of the same step? If there was only one centrifugation, please remove from line 116 for clarity.

**We appreciate the reviewer's request for clarification on this point.**

**The centrifugation step in line 109 refers to the samples that undergo filtration.**
**The centrifugation step in line 118 refers to the non-filtered samples.**

**We amended line 109 to read: “Before the filtration step, 40mL of the composite WW samples selected for filtration were centrifuged at 3000 x g for 10 minutes to remove solids.”**

- Line 142 – please give final concentration of RP primer or include stock concentration used for dilution.

**The concentration of RP primer is not indicated by the company (line 144).**

- Line 145 – please remove repetition of “reaction”.

**We removed the repetition in line 147.**

- Line 153 – please state the unit of concentration (ng/ul?).

**The unit of concentration in line 155 is ng/µL.**

- Line 165 – please clarify whether one million PE reads was the aim for sequencing depth or whether this normalisation was achieved during bioinformatic analysis.

**In line 167 the sentence reads: “One million PE was the aim of sequencing depth for each sample.”**

- Line 177 – please detail which programs were used for trimming and consensus genome generation.

**The software used for trimming and consensus genome generation is CLC genomic workbench. This has been specified from line 182 in paragraph “Bioinformatic analysis”.**

- Line 180 – Please clarify what “minimum frequency” refers to – possibly minimum frequency of variations?

**We clarify “minimum frequency” in line 185 with this sentence: *“the minimum frequency of nucleotide variation was set at 3%”*. The sentence now reads: *“As configurable parameters, the minimum frequency of nucleotide variation and the minimum average base quality were set at 3% and 25.0 respectively.”***

- Lines 194-199 – section “as detailed…followed by purification and concentration” not required as this is already described in the methods section.

**We have removed this section in paragraph 3.1 “Sample preparation”.**

- Figure 1 and Table S2 – please indicate whether there were any significant differences between treatments in these metrics.

**The significant differences are now indicated in the caption of Figure 1, lines 253-261.**

- Line 235-236 – please clarify what is meant by “in intra- and inter-conditions”.

**Intra-condition means within each condition (e.g. WWTP1, WWTP2, WWTP3 for F-T condition), whereas inter-condition means between conditions (e.g. WWTP1 for F-NT, F-T, NF-NT, NF-T). We amend the sentence that now reads: “Moreover, the number of viral cp input into library preparation was not identical in intra- (within) and inter (between) -conditions,”. Lines 248-249.**

- Figures 1/3/4/5/6 – These plots are referred to as histograms, when they are bar plots with categorical variables on the x-axis.

**Thank you for the clarification. It has been corrected with bar plots.**

- Figure 1 – Consider replacing this with a plot representing the data in Table S2, as this is what makes up the majority of results discussed in this section.

**We believe that Figure 1 better illustrates the point of this section because it describes the viral copies that are loaded into the library preparation.**
**The viral copies input were statistically significantly lower in the F-NT condition compared to the NF conditions, but the F-NT condition shows a better sequencing result.**

- Figure 2 – The colours in this figure are not clear, probably due to the low resolution of the image which also makes the axis labels illegible.

**We have uploaded a higher-resolution image.**

- Line 259 – Please clarify what is referred to as “lower lines”.

**In this paragraph, we are describing the electropherogram peaks at 60 seconds. Those peaks are unspecific sequences like primer dimers. With “lower lines” at the 60-second frame, we are referring to the group of lines that appear just over the baseline but are lower than the counterpart higher lines. We amend the word “lines” with “peaks” in line 275.**

- Line 263 – It is unclear what is meant by “genomic contamination” and how this would impair sequencing – please discuss further to clarify.

**We clarified the sentence from line 278: “In addition, Trujillo et al. argue that the removal of solids by filtration facilitates downstream processing steps by eliminating genomic contamination, such as nucleic acid from other species, that could be unspecifically amplified and might hinder the ability to deeply sequence SARS-CoV-2.”**

- Line 283 – it is unclear whether all samples had a percentage of reads classified as SARS-CoV-2 of less than 40%, or just the means for these treatments are <40%.

**The means of the percentages for each treatment are <40%.**

**We amended the sentence in lines 300-303:” The percentage of the means of the raw reads classified as belonging to SARS-CoV-2 is shown in Fig 3A. In the F-NT samples, 62% ± 7 of the means of the reads were classified as SARS-CoV-2; whereas, in the F-T, NF-NT and NF-T samples, only ≤40% of the means of the reads were classified as aligned to SARS-CoV-2".**

- Line 289 – Please explain further the speculation that RNA sample concentration would lead to increased bacterial reads.

**We thank the reviewer for making this point.**

**After reviewing the statistics, we have removed the sentence about the concentration step being the reason for the increase of bacteria reads %.**

- Line 296-297 – Could you provide some discussion as to why wastewater filtration may improve quality scores?

**Lines 318-324: The F-NT samples show a higher quality score compared to the other conditions. This may be due to the fact that the filtration removes off-target sequences that could be randomly amplified during the library process and generate unspecific and short sequences. The generation of unspecific and short sequences has been detected in the electropherograms in Figure 2A-B. Specifically for Illumina sequencing (Sequencing By Synthesis technology), these unspecific and short reads could negatively affect flow cell saturation, clustering formation and base call quality score as, especially for short sequences, if the sequencing read length is longer than the library insert size, sequencing may continue through the Rd1SP-Rd2SP (Read 1 and 2 Sequencing Primer), index and adapter sequences, resulting in a decrease in quality score.**

**We add the following sentence in line 318: “This may be due to the fact that the WW filtration removes off-target sequences that could be randomly amplified during the library process and generate unspecific and short sequences, as also seen in Figure 2. Specifically for Illumina sequencing (Sequencing By Synthesis technology), these unspecific and short reads could negatively affect flow cell saturation, clustering formation and base call quality score as, especially for short sequences, if the sequencing read length is longer than the library insert size, sequencing may continue through the Rd1SP-Rd2SP (Read 1 and 2 Sequencing Primer), index and adapter sequences, resulting in a decrease in quality score.”**

- Section 3.4.2.2 – The percentage of reads assigned to SARS-CoV-2 was much higher with CLC workbench than Kraken. It would be good to comment on why this is, possibly due to raw reads used in Kraken classification including many short reads which are filtered out before direct mapping.

**The difference in the percentage of reads classified as belonging to SARS-CoV-2 using Kraken and the percentage of SARS-CoV-2 mapped reads using CLC Genomic Workbench can be mainly attributed to the differences in the input files and also to the methodologies and algorithms used by these tools for classification and mapping. As input file, Kraken pipeline uses fastq file that are raw reads, whereas CLC workbench pipeline works with reads that are already filtered.**

**We add the following comment (lines 350-353) “The percentage of reads assigned to SARS-CoV-2 was much higher for all conditions using the CLC genomic workbench software than the Kraken pipeline, possibly because the raw reads used in Kraken classification include many short reads that are filtered out before direct mapping using the CLC genomic workbench.”**

- Figure S2 is redundant as the % reads not mapped can be extrapolated from % mapped reads.

**Figure S2 (% of not mapped reads) has been removed from the manuscript. Figure 5 (% of mapped reads) has been moved as figure S2.**

- Line 343 – The difference in % mapped reads is only significant between F-NT and F-T, but not between NF-NT and NF-T. **After reviewing the statistics, the figure describing the percentage of mapped reads was not significant. It has been moved to the supporting information section as figure S2.**

- Line 347 – amend “off-target reads” as these are not reads at this point e.g off-target nucleic acid fragments.

**We amended every sentence in which this error was made.**

- Line 346-347 – This finding is contrary to Mondal et al 2021 who found RNA purification and DNase treatment to increase % reads aligned to SARS-CoV-2, and Child et al 2023 (<https://doi.org/10.1371/journal.pone.0284211>) who found RNA purification and concentration to also increase alignment rate. Please discuss your findings in relation to these studies.

**We appreciate the reviewer's request to discuss this important point further.**

**The three studies (our study, Mondal et al., 2021 and Child at al., 2023) were performed under different experimental conditions. First, the nucleic acid extraction kits were the same for this paper and Mondal et al. 2021, but different fro Child et al. 2023. Second, the three papers used different library preparation kits. Third, the tools used for the bioinformatics analysis were different. These experimental differences could affect the sequencing results in terms of % reads aligned to SARS-CoV-2 and alignment rate. As we mention in lines 466-469, an appropriate SARS-CoV-2 sequencing result from WW could benefit from RNA purification, concentration and DNase treatment. In our opinion, the RNA treatment should be carried out, upon WW filtration, based on the experimental scenario, in relation to the type of extraction kit, library preparation kit and analysis tools used.**

- Line 375 – amend “essential” as low frequency variants are still identified in unfiltered samples.

**We amended the word “essential” with “increase” in lines 424-425: “These results suggested that the WW filtering step increases the detection of very low-frequency mutations, allowing for the monitoring of merging variants”.**

- Line 388-9 – Could you provide statistical tests to back up the statement that “mutation frequency was higher in the F conditions”? Also, please discuss why the mutation frequency would be higher in filtered samples if these mutations are associated with the BA.1 variant, which is stated to be dominant in the samples. Could the unfiltered treatments be picking up on more diversity of variants than filtered treatments, reducing the frequency of these typical BA.1 mutations in favour of other variants?

**We thank the reviewer for this point of view. We have not performed any statistical tests on the frequency of BA.1 mutations as 6 or 7 out of 10 mutations relative to the non-filtered conditions (either treated or not) do not meet the 30X coverage requirement. Therefore, it is not possible to recover a frequency for these mutations for NF-NT and NF-T samples.**

**To answer the question “if the unfiltered treatment could be picking up on more diversity of variants than filtered treatments reducing the frequency of these typical BA.1 mutations in favour of other variants” we think that since the BA.1 is the dominant variant in all the three WWTPs, its mutations should at least be detected at low frequency. In the experiment of Fig 6, the analyzed spike area didn’t reach the coverage ≥30X, which means that the number of reads generated was low, possibly due to the low-quality of the viral input RNA.**

- Line 402 – Please amend this statement, as only the F-NT treatment passed for all samples, not F-T.

**We thank you for this comment. We amended the statement in line 456 removing the F-T sample.**

- Figure 8 – The colour scale used in this figure requires clarification. These percentages are clearly not directly related to the percentage parameters in the “Sequencing parameters” column. Is this scale relative to the highest and lowest value for each parameter amongst the treatments?

**Yes, the color scale is relative to the highest and lowest value for each parameter among the treatments. Figure 8 becomes Figure 7. We added the sentence in lines 499-500.**
