# Supplementary material for: Efficient wastewater sample filtration improves the detection of SARS-CoV-2 variants: An extensive analysis based on sequencing parameters
Source: PLoS One. 2024 May 24;19(5):e0304158. doi: 10.1371/journal.pone.0304158 (PMC11125551; doi:10.1371/journal.pone.0304158)
Supplement: Attachment — Submitted filename: Response to Reviewers.docx [file pone.0304158.s008.docx]

**Dear Dr. Poovendhree,**

**Thank you for your letter and the opportunity to submit the revised manuscript. We have addressed all the points raised by the editor’ and reviewers’ comments (bold sentences). Each point has been addressed, with changes tracked in the accompanying manuscript. We appreciate the chance to revise and submit our manuscript.**

Additional Editor Comments:

Please ensure that the quality of your figures is improved in the next revision.

**The quality of the figures has been checked and improved through the PACE digital diagnostic tool.**

Reviewers' comments:

Reviewer #2: The authors have conducted a study comparing methods for preparation of wastewater samples for amplicon sequencing of SARS-CoV-2, in particular assessing how the quality of sequencing data is impacted by prefiltering wastewater and implementing a step for purifying and concentrating extracted RNA. The authors conclude that filtration of wastewater samples leads to improved specificity and coverage of SARS-CoV-2 sequencing data, while treatment of RNA samples reduced the percentage of sequencing reads aligned to SARS-CoV-2 but had little impact on other sequencing metrics.
The experimental design and metrics used to assess SARS-CoV-2 sequencing quality are valid, and following amendments in response to the initial round of reviewers comments the statistical methods used are now valid and clear. Considering this, I recommend that this manuscript requires only minor revisions. I understand that these are largely grammatical suggestions, but I hope the authors appreciate the improvement in clarity that they can provide.

Minor Issues
- Figure legends – Two way ANOVA results are currently givenm in the format “Two-way ANOVA (* = p ≤ 0.05).”. Please amend these without the asterisk as otherwise it seems to be referring to the asterisks in the plot which are the paired T test results e.g. “Two-way ANOVA (p ≤ 0.05)”.

**We thank the reviewer for this appropriate comment. It helps to improve the clarity of the result. We have amended all the sentences in which the ANOVA result was stated.**

- Figure 2 – The resolution of Figure 2 is still very low in the version I have been sent. This should be checked before publication.
**The resolution of Figure 2 has been improved.**

- Line 54 – Suggested revision of “Wastewater Based Epidemiology (WBE) was proposed as an epidemiological tool approach for wastewater (WW) surveillance” to “Wastewater Based Epidemiology (WBE) was proposed as an epidemiological tool approach for disease surveillance”
**We have amended the sentence. We have added the word “wastewater” before “WW” in line 57.**

- Line 59 – “asymptomatic host” needs amending to “asymptomatic hosts”
**We have amended the word.**

- Line 80-83 – Suggested revision of “However, most of the viral genome sequences used to monitor viruses’ evolution have been“ to “However, most of the viral genome sequences used to monitor the evolution of viruses have been“, and “but not many monitoring have been performed” to “but not much monitoring has been performed”
**We have amended the sentences.**

- Line 87-89 – please amend to “Earlier than the development of WBE for the COVID-19 pandemic, previous studies showed that the detection of viral pathogens in sewage depended on the concentration and extraction methods used (21–23).”
**We have amended the sentence.**

- Line 248-249 – may I suggest an amendment to “Moreover, the number of viral cp input into library preparation was not identical within or between conditions...”

**We have amended the sentence.**
